# Weekend discharge after hip fracture surgery is associated with increased 30-day mortality. A retrospective observational study of 35,138 hip fractures reported to the Norwegian Hip Fracture Register

**DOI:** 10.1007/s41999-025-01329-2

**Published:** 2025-10-16

**Authors:** Andrea Toft Boutera, Eva Dybvik, Geir Hallan, Torbjørn Berge Kristensen, Jan-Erik Gjertsen

**Affiliations:** 1 Department of Orthopedic Surgery, Diakonhjemmet Hospital, Oslo, Norway; 2 Department of Clinical Medicine, University of Bergen, Bergen, Norway; 3 The Norwegian Hip Fracture Register Department of Orthopedic Surgery, Haukeland University Hospital, Bergen, Norway; 4 Department of Orthopedic Surgery, The Coastal Hospital at Hagevik, Haukeland University Hospital, Bergen, Norway

**Keywords:** Hip fracture, Discharge from hospital, Mortality, Readmission risk

## Abstract

**Purpose:**

The influence of discharge timing after hip fracture surgery has been sparsely investigated. We aimed to investigate whether time of discharge influence 30-day mortality, and secondarily 1-year mortality and 30-day readmission risk after hip fracture surgery.

**Methods:**

Data from 35,138 hip fracture patients aged ≥ 65 and with length of stay (LoS) ≤ 14 days reported to the Norwegian Hip Fracture Register 2008 to 2018 were analyzed. Cox regression analyses, adjusted for age groups, sex, ASA class, cognitive function, fracture type, and type of surgery were used to calculate mortality risk after discharge on weekdays compared to weekends (Friday 18:00 to Monday 08:00). Patients were grouped by age, cognitive function, ASA class, and discharge destination.

**Results:**

Mean age was 83 years, 71% were women, and 61% had ASA class ≥ 3. Patients discharged on weekends were marginally older, more often ASA class ≥ 3, cognitively impaired, and had shorter LoS than patients discharged on weekdays. Weekend discharge was associated with increased 30-day mortality (HRR 1.4, 95% CI 1.3–1.6; *p* < 0.001) and 1-year mortality (HRR 1.2, CI 1.2–1.3; *p* < 0.001) compared to weekday discharge. Sub-analyses showed increased 30-day mortality for all age groups except 65–74 years, all ASA groups, patients with and without cognitive impairment, patients discharged to nursing homes and the most common surgical methods. No statistically significant difference in 30-day readmission risk was found after weekend discharged compared to weekday discharge.

**Conclusion:**

Weekend discharge for hip fracture patients was associated with increased 30-day and 1-year mortality, but did not influence overall 30-day readmission risk.

## Key summary points

**Aim:**

To investigate whether discharge time from hospital influences mortality and readmission risk after hip fracture surgery.

**Findings:**

Discharge on weekends was associated with increased 30-day and 1-year mortality, but no difference in 30-day readmission risk compared to discharge on weekdays.

**Message:**

Our study highlights the need for enhanced attention during weekend discharges of hip fracture patients.

## Introduction

A hip fracture is a frequent cause of acute surgery, and the most common and severe low-energy fracture among older people admitted to hospital [1]. The after hip fractures in older patients is 8%, and 1-year mortality as high as 24% [2]. Furthermore, hip fractures have a severe impact on both patients and society, leading to an increased need for institutionalization, with only 30% of patients regaining their previous functional level [3].

Several studies have explored how the timing of admission and surgery affect outcome after a hip fracture [4–7]. The influence of the timing of discharge, on the other hand, has been less described in the literature. Two earlier studies used data from The Norwegian Patient Registry and reported increased mortality for hip fracture patients discharged from hospitals on weekends and holidays compared to weekdays [8, 9]. These studies had, however, no data on time of fracture, time of surgery, discharge destination, comorbidity, or readmissions. In Norway, the number of hospital beds has been reduced over the last decades, from 22,000 in 1980 to less than 11,000 in 2020 [10, 11]. Accordingly, there are increasing demands for early discharge of hip fracture patients, also over the weekends. In Norway, patients with hip fractures are most often discharged to a short-term or permanent nursing home placement; they may also return to their own homes, with or without municipal home nursing services, and only rarely are they transferred to rehabilitation wards or other institutional facilities [12]. The organization of the Norwegian healthcare system makes it more difficult to establish support services for patients in primary care during the weekend. Thus, it may be easier to discharge patients living permanently in a nursing home over the weekend than those in need of primary care support service. Ensuring safe discharge from the hospital for hip fracture patients will have important quality and health policy implications. Identifying whether the time of discharge affects the risk of mortality and readmissions is of great value.

Having access to data on patient characteristics through the Norwegian Hip Fracture Register (NHFR) and discharge details from the Norwegian Patient Registry (NPR), the aim of this study was to investigate if time of discharge influences 30-day mortality, and secondarily 30-day readmission risk after hip fracture surgery.

## Methods

### Study design

**Fig. 1:**
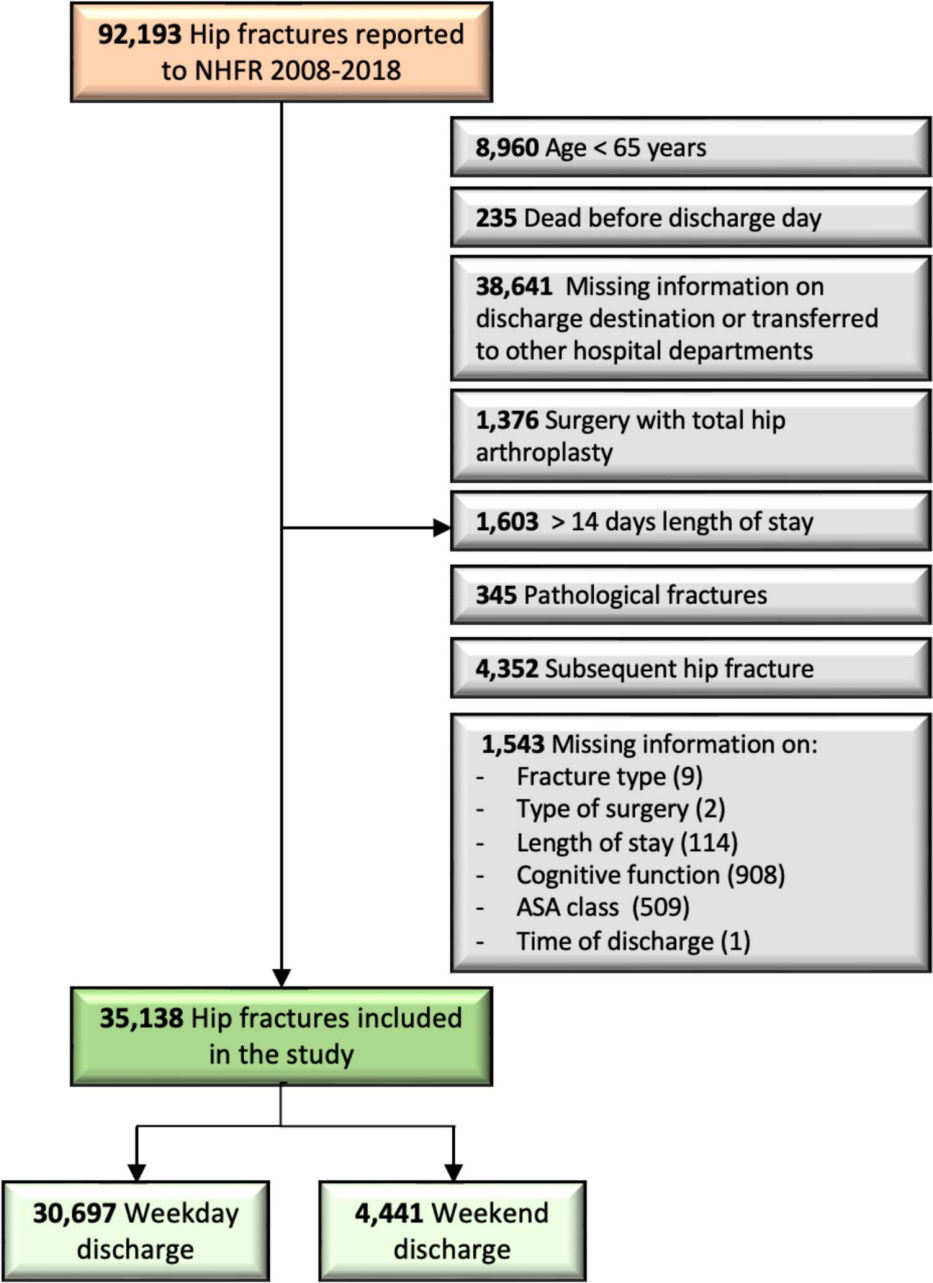
Flowchart for patients. *NHFR* Norwegian Hip fracture Register*, ASA* American Society of Anesthesiology

In this retrospective observational study, we used prospectively collected data from the NHFR, which includes data on hip fracture surgery performed in Norwegian hospitals since 2005 [13]. Using a standardized questionnaire, the surgeons report information about the fracture, the patient, and the surgery to the NHFR. The completeness of primary surgery within the NHFR has been found to be 86% for osteosyntheses and 92% for hemiarthroplasties [2]. The Norwegian personal identification number facilitates prospective follow-up of the individual case and relates to data from the Norwegian Patient Registry (NPR). The NPR has information on the discharge time, discharge destination, and readmissions. The data from NPR has been personally identifiable since 2008 [14] and has been linked to NHFR until 2018. We, therefore, used the available data from 2008 to 2018 consisting of 92,193 hip fractures reported to both the NHFR and the NPR (Fig. 1).

We included all patients ≥ 65 years with non-pathological hip fractures. Patients who died before the discharge day, and patients transferred to other hospital departments were excluded. Patients with a length of stay (LoS) > 14 days were also excluded, as this is significantly longer than the average LoS of 5 days for hip fracture patients in Norway, and because a severely prolonged LoS may indicate that there have been medical complications that may influence both mortality and readmission risk. Furthermore, we excluded subsequent hip fractures during the follow-up, ensuring that each patient could only contribute with one hip fracture in the analyses. Hip fracture patients operated with total hip arthroplasty are reported to the Norwegian Arthroplasty Register (NAR). The NAR lacks information on cognitive function, fracture type and time of surgery. Since these are essential variables in this study and are included as covariates in the Cox regression analyses, we also chose to exclude these patients.

Finally, we excluded hip fracture patients with missing data on discharge day, discharge time, discharge destination, fracture type, type of surgery, cognitive function, and ASA class.

The final dataset consisted of 35,138 hip fracture patients, and of these 30,697 was discharged on a weekday and 4441 on weekends (Fig. 1).

Comorbidity was classified using the American Society of Anesthesiology (ASA) physical status by the anesthesiologists, and cognitive impairment was classified as “yes”, “no”, or “uncertain” based on information from the medical report or from relatives. Discharge destinations were categorized into four groups: Home, Nursing home, Rehabilitation, and Other. In the NPR, the category “Home” consisted of patients who lived permanently in a private residence, as well as some patients who prior to admission lived permanently in a nursing home. In contrast, the category “Nursing home” included exclusively patients who were discharged to a nursing home, but prior to the fracture, these patients may have been permanent nursing-home residents or may have lived independently in their own homes. Weekend was defined as Friday 18:00 to Monday 08:00. All other days were referred to as weekdays. For sub-analyses, patients were stratified into 3 ASA class groups (1–2, 3, and 4–5), 5 age groups (65–74, 75–79, 80–84, 85–89 and ≥ 90 years), and groups based on cognitive impairment (yes, no, or uncertain).

### Statistical analysis

Categorical variables were compared using the chi-square test. Two-sided student´s t-test was used to estimate *p*-value for the mean age. Survival analyses were performed using Cox regression and Kaplan–Meier analyses. Cox regression analyses were used to compare hazard rate ratios (HRRs) of death within 30-days and 1-year from surgery between discharge on a weekend and discharge on a weekday. HRRs are presented with 95% confidence intervals (CIs).

**Fig. 2:**
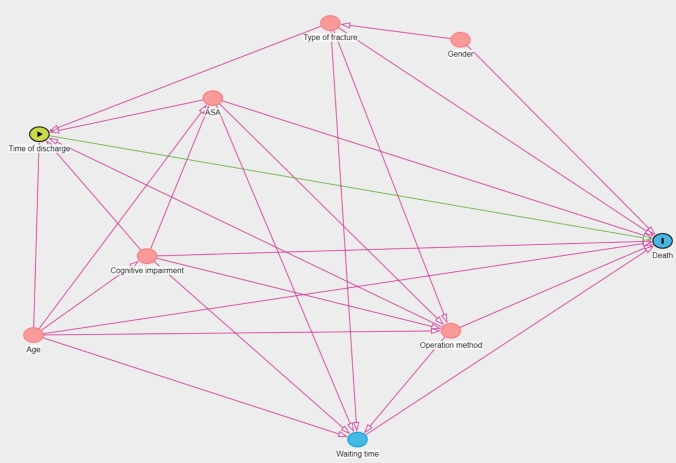
DAGitty showing variables that needed to be adjusted for in the Cox model. *ASA* American Society of Anesthesiology

We used the free program DAGitty (www.dagitty.net) version 3.1 (2023) to evaluate variables that needed to be adjusted for in the Cox model. We developed directed acyclic graphs (DAGs) for mortality after hip fracture (Fig. 2). According to this model, Cox regression analyses for time of discharge were adjusted for sex, age groups, ASA class, cognitive impairment, fracture type, and type of surgery. The follow-up time was calculated from the time of surgery to death or 30-days postoperative. Proportionality assumption was checked using a log minus log plot and was fulfilled. Readmissions within 30-days after surgery for weekend discharge and weekday discharge were calculated using the chi-square test. Readmissions were calculated from the time of discharge to 30-days after surgery. A readmission was defined as any cause of hospital readmission, including readmissions due to a subsequent contralateral hip fracture.

Dates of death were identified from files provided by Norwegian Population Register. Statistical significance was defined as *p*-values < 0.05. All statistical analyses were preformed using IMB-SPSS Statistics, version 29.0 for Windows (IBM Corp, Armonk, NY, USA), and the statistical package R, version 4.2.3 (http://www.R-project.org). Our study was performed in accordance with the RECORD Statement.

## Results

The study included 35,138 hip fracture patients with a mean age of 83, and 71% were female. Among these, 30,697 (87%) patients were discharged on weekdays, while 4,441 (13%) were discharged on weekends (Table 1). Most patients (61%) had severe comorbidity (ASA class ≥ 3). Minor differences in fracture type and surgery method were observed in patients discharged on weekends compared to weekdays. Patients discharged on weekends were marginally older, had more often ASA class ≥ 3, were more often cognitively impaired, and had shorter length of stay (LoS) than patients discharged on weekdays. Patients discharged to a nursing home were older, had more comorbidity, and were more often cognitively impaired (Table 2). Mean LoS was 6 days for weekday discharges and 4 days for weekend discharges both when investigating the whole study population and patients discharged to a nursing home exclusively.

**Table 1:** Baseline characteristics of all patients

	Discharge		
	Weekday	Weekend	*p*-value
Total (%)	30,697 (87)	4441 (13)	
Age category, *n* (%)			< 0.001^a^
65–74	5525 (18)	780 (18)	
75–79	4653 (15)	583 (13)	
80–84	6538 (21)	913 (21)	
85–89	7709 (25)	1114 (25)	
≥ 90	6272 (20)	1051 (24)	
Mean age, (SD)	82.6 (7.9)	83.1 (8.1)	< 0.001^b^
Female, *n* (%)	21,908 (71)	3143 (71)	0.412^a^
ASA classification, *n* (%)			< 0.001^a^
ASA 1–2	12,042 (40)	1508 (34)	
ASA 3	16,879 (55)	2614 (59)	
ASA 4–5	1776 (6)	319 (7)	
Cognitive impairment, *n* (%)			< 0.001^a^
Yes	7887 (26)	1870 (42)	
No	20,108 (66)	2243 (51)	
Uncertain	2702 (9)	328 (7)	
Type of fracture, *n* (%)			0.003^a^
Undisplaced FNF	4548 (15)	718 (16)	
Displaced FNF	13,211 (43)	1789 (40)	
Basocervical FNF	817 (3)	115 (3)	
Trochanteric AO/OTA A1	4757 (16)	765 (17)	
Trochanteric AO/OTA A2	5002 (16)	733 (17)	
Trochanteric AO/OTA A3	694 (2)	89 (2)	
Subtrochanteric	1454 (5)	198 (5)	
Other	214 (1)	34 (1)	
Surgical method, *n* (%)			0.006^a^
Screw osteosynthesis	5029 (16)	789 (18)	
Hemiarthroplasty	12,807 (42)	1726 (39)	
Sliding hip screw	8047 (26)	1196 (27)	
Short IM nail	3013 (10)	456 (10)	
Long IM nail	1801 (6)	274 (6)	
Waiting time to surgery, *n* (%)			0.002^a^
0–6 h	1183 (4)	147 (3)	
> 6–12 h	4315 (14)	621 (14)	
> 12–24 h	11,197 (37)	1570 (35)	
> 24–48 h	9091 (30)	1300 (29)	
> 48 h	4358 (14)	730 (16)	
Missing	552 (2)	73 (2)	
Discharge destination, *n* (%)			< 0.001^a^
Home	12873 (42)	1,843 (42)	
Nursing home	14,106 (46)	2104 (47)	
Rehabilitation	782 (3)	66 (2)	
Other	2936 (10)	428 (10)	
Mean length of stay, (SD)	5.6 (2.8)	4.3 (2.4)	< 0.001^b^

*n* Total number, *SD* Standard deviation, *ASA* American Society of Anesthesiology, *FNF* Femoral neck fracture, *AO/OTA* Arbeitsgemeinschaft für Osteosynthesefragen/Orthopaedic Trauma Association, *IM* intramedullary

^a^Student’s t-test

^b^Pearson Chi-squared test, n

**Table 2:** Baseline characteristics of patients discharged to nursing home

	Discharge to nursing home		
	Weekday	Weekend	*p*-value
Total (%)	14,106 (87)	2104 (13)	
Age category, *n* (%)			0.079^a^
65–74	1411 (10)	188 (9)	
75–79	1786 (13)	240 (11)	
80–84	3096 (22)	454 (22)	
85–89	4138 (29)	625 (30)	
≥ 90	3675 (26)	597 (28)	
Mean age, (SD)	84.5 (7.1)	85.1 (7.1)	< 0.001^b^
Female, *n* (%)	10,188 (72)	1529 (73)	0.669^a^
ASA classification, *n* (%)			< 0.001^a^
ASA 1–2	4492 (32)	555 (26)	
ASA 3	8680 (62)	1,385 (66)	
ASA 4–5	934 (7)	164 (8)	
Cognitive impairment, *n* (%)			< 0.001^a^
Yes	4548 (32)	1078 (51)	
No	8098 (57)	834 (40)	
Uncertain	1460 (10)	192 (9)	
Type of fracture, *n* (%)			0.009^a^
Undisplaced FNF	1566 (11)	272 (13)	
Displaced FNF	6125 (43)	868 (41)	
Basocervical FNF	378 (3)	62 (3)	
Trochanteric AO/OTA A1	2272 (16)	387 (18)	
Trochanteric AO/OTA A2	2575 (18)	344 (16)	
Trochanteric AO/OTA A3	371 (3)	47 (2)	
Subtrochanteric	708 (5)	105 (5)	
Other	111 (1)	19 (1)	
Surgical method, *n* (%)			0.076^a^
Screw osteosynthesis	1673 (12)	291 (14)	
Hemiarthroplasty	6055 (43)	853 (41)	
Sliding hip screw	4006 (28)	604 (29)	
Short IM nail	1479 (11)	225 (11)	
Long IM nail	893 (6)	131 (6)	
Waiting time to surgery, *n* (%)			0.087^a^
0–6 h	422 (3)	48 (2)	
> 6–12 h	1844 (13)	257 (12)	
> 12–24 h	5128 (36)	757 (36)	
> 24–48 h	4379 (31)	652 (31)	
> 48 h	2055 (15)	350 (17)	
Missing	278 (2)	40 (2)	
Mean length of stay, (SD)	5.6 (2.8)	4.1 (2.3)	< 0.001^b^

*n* number, *SD* Standard deviation, *ASA* American Society of Anesthesiology, *FNF* Femoral neck fracture, *AO/OTA* Arbeitsgemeinschaft für Osteosynthesefragen/Orthopaedic Trauma Association, IM Intramedullary

^a^Student’s t-test

^b^Pearson Chi-squared test, *n*

### Mortality

**Fig. 3:**
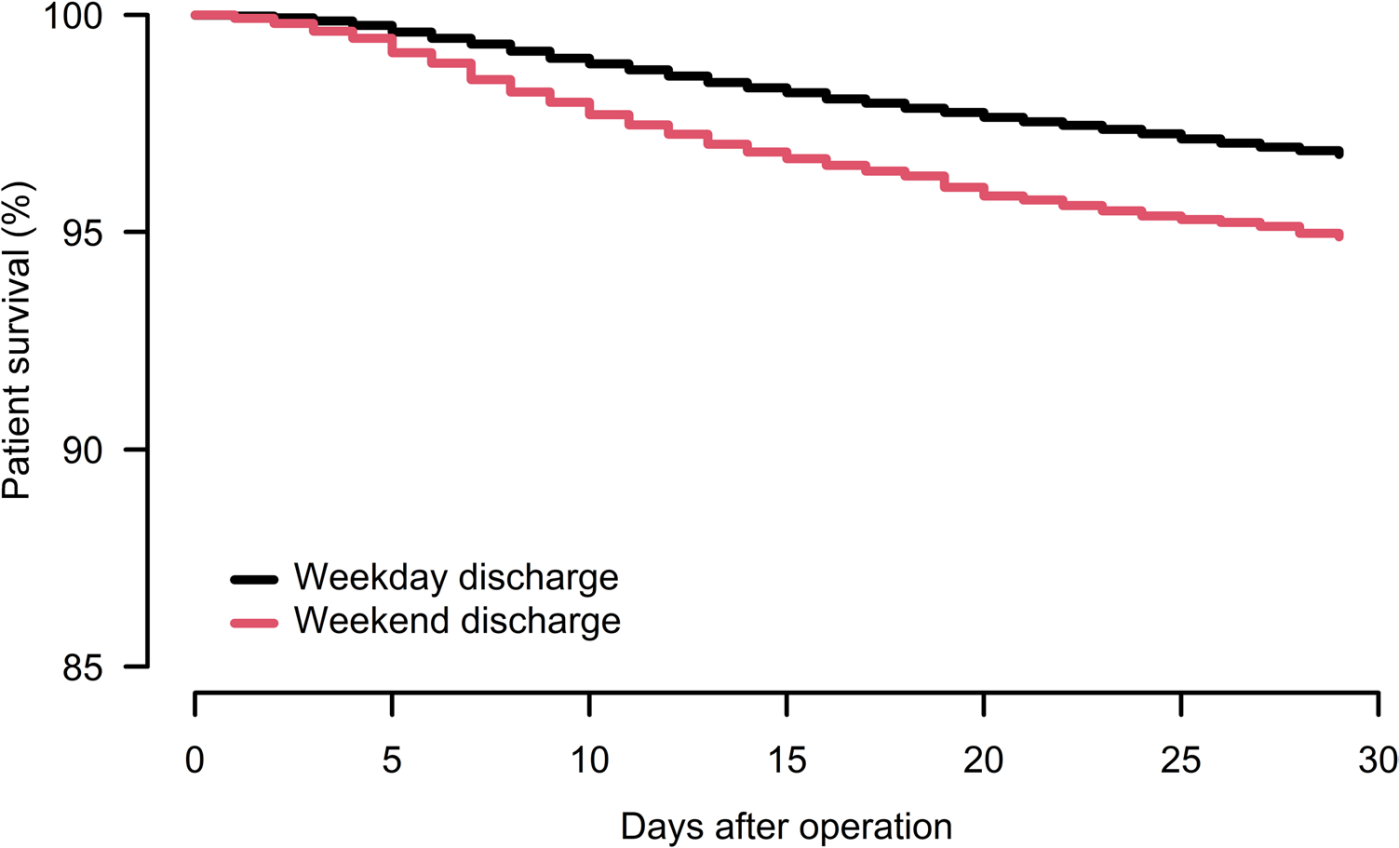
30-day survival after discharge for all 35,138 patients. Cox analyses adjusted for age groups, sex, ASA class, cognitive impairment, fracture type and type of surgery

The 30-day mortality was 5.6% (1952 out of 35,138 patients) in the whole study population, 5.0%% (1545 out of 30,697 patients) after weekday discharge and 9.2% (407 out of 4441 patients) after weekend discharge. Discharge on weekends was associated with increased 30-day mortality (HRR 1.4, CI 1.3–1.6; *p* < 0.001) compared to weekdays (Fig. 3). Sub-analyses showed that both healthy patients (ASA 1–2) and comorbid patients (ASA 3 and ASA 4–5) had increased 30-day mortality when discharged on weekends compared to weekdays (Table 3). Further, patients in all age groups except 65–74 years had increased 30-day mortality after weekend discharge. The increased 30-day mortality after weekend discharge was found for both cognitively impaired and cognitively fit patients (Table 3). When including only patients discharged to a nursing home, weekend discharge was associated with increased 30-day mortality compared to weekday discharge (5.5% vs 13%, HRR 1.3, CI 1.12–1.51; *p* < 0.001) (Table 3). Finally, when investigating 30-day mortality based on surgical method, we found that weekend discharge was associated with a significant increase in mortality among patients who were treated with hemiarthroplasty, sliding hip screw and short intramedullary nail during weekends compared to weekday discharge (Table 3).

**Table 3:** 30-day mortality in all patients and in subgroups. Cox regression analysis calculating hazard rate ratio for weekend discharge compared to weekday discharge

	Weekday		Weekend			
	N death	HRR	N death	HRR	95% CI	*p*-value^a^
All patients:	1545	1 (ref)	407	1.4	1.30–1.60	< 0.001
Age groups:						
65–74 years	85	1 (ref)	13	0.86	0.50–1.60	0.623
75–79 years	133	1 (ref)	34	1.5	1.04–2.20	0.031
80–84 years	260	1 (ref)	85	1.8	1.40–2.30	< 0.001
85–89 years	447	1 (ref)	107	1.4	1.10–1.70	0.006
≥ 90 years	620	1 (ref)	168	1.4	1.17–1.65	< 0.001
ASA class:						
1–2	193	1 (ref)	54	1.6	1.15–2.12	0.004
3	1041	1 (ref)	274	1.4	1.24–1.62	< 0.001
4–5	311	1 (ref)	79	1.3	1.03–1.70	0.031
Cognitive impairment:						
Yes	930	1 (ref)	287	1.3	1.17–1.52	< 0.001
No	448	1 (ref)	88	1.7	1.37–2.17	< 0.001
Uncertain	167	1 (ref)	32	1.5	1.00–2.13	0.053
Surgical method:						
Screw osteosynthesis	190	1 (ref)	51	1.2	0.91–1.69	0.181
Hemiarthroplasty	612	1 (ref)	160	1.6	1.31–1.86	< 0.001
Sliding hip screw	504	1 (ref)	129	1.3	1.11–1.63	0.003
Short IM nail	153	1 (ref)	44	1.6	1.13–2.23	0.008
Long IM nail	86	1 (ref)	23	1.3	0.80–2.03	0.318
Discharge destination:						
Nursing home	894	1 (ref)	214	1.3	1.12–1.51	< 0.001

*N* Number, *ASA* American Society of Anesthesiology, *IM* Intramedullary,

*HRR* Hazard rate ratio, *CI* Confidence interval

^a^Cox regression analysis adjusted for age groups, sex, ASA groups, cognitive function, type of fracture and type of surgery

**Fig. 4:**
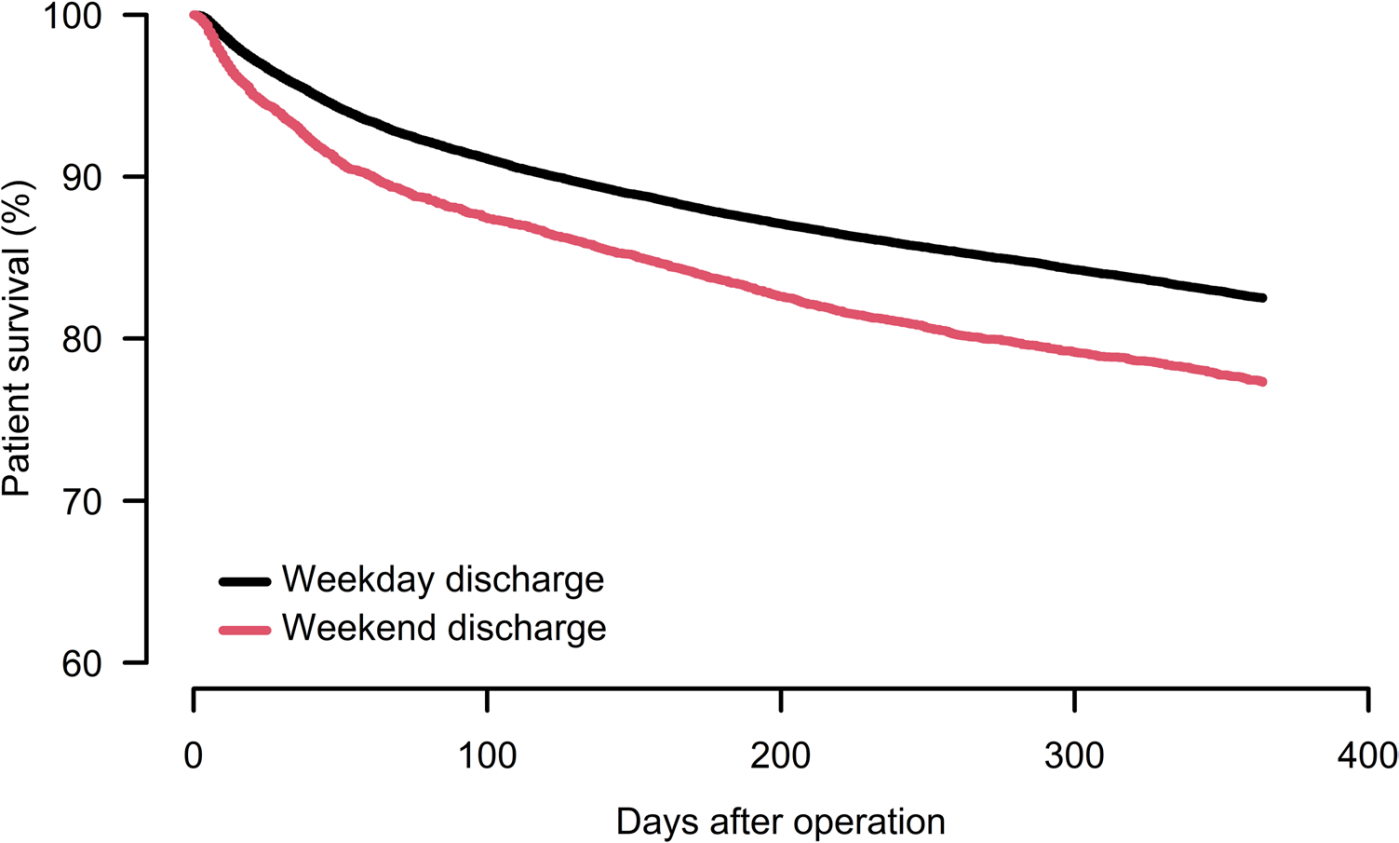
1-year survival after discharge for all 3*5,138* patients. Cox analyses adjusted for age groups, sex, ASA class, cognitive impairment, fracture type and type of surgery

The 1-year mortality was 23% (7933 out of 35,138 patients) in the whole study population, 21% (6580 out of 30,697 patients) after weekday discharge and 30% (1353 out of 4,441 patients) after weekend discharge. Discharge on weekends was associated with increased 1-year mortality (HRR 1.2, CI 1.2–1.3; *p* < 0.001) compared to weekdays (Fig. 4).

### Readmissions

There was no statistically significant nor clinically important difference in 30-day readmission risk between patients discharged on weekends compared to those discharged on weekdays (15% vs 16%). When looking at subgroups of patients, we found that weekend discharge of patients 75–79 years old was associated with an increased 30-day readmission risk compared to weekday discharge. On the other hand, weekend discharge of patients 80–84 years old, cognitively impaired patients, and patients operated with screw osteosynthesis was associated with a lower 30-day readmission risk compared to weekday discharge (Table 4).

**Table 4:** Readmissions within 30 days after surgery

	Weekday	Weekend	*p*-value^a^
	N readmitted	N readmitted	
All patients, *n* (%)	4942 (16)	679 (15)	0.169
Age groups:			
65–74 years	710 (13)	92 (12)	0.407
75–79 years	700 (15)	114 (20)	0.005
80–84 years	1133 (17)	134 (15)	0.046
85–89 years	1360 (18)	175 (16)	0.112
≥ 90 years	1039 (17)	164 (16)	0.436
ASA class:			
1–2	1449 (12)	175 (12)	0.629
3	3115 (19)	441 (17)	0.051
4–5	378 (21)	63 (20)	0.536
Cognitive impairment:			
Yes	1368 (17)	278 (15)	0.01
No	3058 (15)	343 (15)	0.916
Uncertain	516 (19)	58 (18)	0.537
Surgical method:			
Screw osteosynthesis	617 (12)	73 (9)	0.015
Hemiarthroplasty	2269 (20)	307 (18)	0.943
Sliding hip screw	1313 (16)	184 (15)	0.414
Short IM nail	464 (15)	69 (15)	0.882
Long IM nail	279 (16)	46 (17)	0.582
Discharge destination:			
Nursing home	2414 (17)	328 (16)	0.082

*N/n* Number, *ASA* American Society of Anesthesiology, *IM* Intramedullary, *HRR* Hazard rate ratio, *CI* Confidence interval

^a^Pearson Chi-squared test

## Discussion

This nationwide registry-based study found that patient discharge on weekends was associated with increased 30-day and 1-year mortality compared to weekday discharge. This finding was statistically significant in all sub-analyses of different patient groups, except patients aged 65–74 years. There was no statistically significant difference in the overall risk of readmission within 30-days between weekend discharge and weekday discharge, but there were minor differences for some subgroups of patients.

Our findings indicate increased mortality for weekend discharge, in line with two earlier Norwegian studies using data from the NPR [8, 9]. Some of the explanations for this could be that those discharged on weekends had somewhat higher ASA class and were more often cognitively impaired compared to patients discharged on weekdays. Both comorbidity and cognitive impairment are factors associated with permanent residency in a nursing home [15]. An earlier observational study of 1010 hip fracture patients 65 years and older categorized the patients into three groups; the relatively fit patients who had sustained an outdoor fall, frail community-dwelling patients who had sustained an indoor fall, and patients from long-term care institutions [12]. For the last group, the authors concluded that nursing care is more important than intensive rehabilitation. Thus, patients living permanently in a nursing home facility may benefit more from early discharge from the hospital instead of orthogeriatric care at the hospital. For community-dwellers, on the other hand, orthogeriatric treatment has been found to improve mobility and ADL [16, 17]. Our experience as clinicians is that patients who are permanent nursing home residents before admission have their beds waiting for them after discharge from the hospital and thus can be more easily discharged on weekends. On the other hand, patients without permanent residency in a nursing home are less likely to be granted a short-term nursing home stay over the weekend and, as such, are less frequently discharged from the hospital on weekends compared to weekdays. However, our results also show that within the group of patients discharged to a nursing home, weekend discharge was associated with increased 30-day mortality compared to weekday discharge. This likely points to the fact that the process of hospital discharge on weekends is complex, and that there are several factors beyond just the discharge destination that influence the outcome. We found increased 1-year mortality across the entire patient population comparing weekend versus weekday discharge, but it is difficult to envision that the timing of hospital discharge in itself would directly impact 1-year mortality. However, complications arising shortly after discharge contribute to increased 30 day mortality and could also lead to a sustained difference in outcomes one year later. On the other hand, a difference in 1-year mortality between patients discharged during weekends versus weekdays may reflect an underlying difference in comorbidity that is not fully accounted for by adjustments for ASA class, sex, age, and cognitive function.

Even when adjusting for differences in sex, age groups, ASA class, cognitive impairment, fracture type, and type of surgery weekend discharge was associated with increased mortality. Based on available data in the present study, we are not able to explain this difference. Guidelines for treatment of hip fractures globally recommend comprehensive evaluation of hip fracture patients’ underlying conditions, investigating the cause of the fall, perform a drug review, establish anti-osteoporosis treatment, evaluating nutritional status, and assessing fall risk [18–20]. Conducting these evaluations likely involves a need for organization, time, and expertise, which are typically more limited during weekends. Proper assessments and interventions are essential for this fragile patient population, as this could help prevent subsequent falls, complications, and reduce the mortality rate. One could therefore speculate if patients discharged on weekends were offered the same evaluation and interventions before discharge as patients discharged on weekdays.

One of the reasons for increased mortality for patients discharged on weekends could be less staffing. At most hospitals, there are less nurses and less physiotherapists working over the weekends compared to weekdays [21]. Specialists, like nutritionist and pharmacist, may not work during the weekends. The surgeon on call might not have been involved in the treatment of the patient and, accordingly, have limited information on the patient at the time of discharge. In addition, the few orthopedic surgeons on call during weekends have a busier time schedule and less time to review patients before discharge compared to weekdays. This may increase the risk for overlooking important information during the discharge, which may lead to an inadequate discharge report. One could also speculate if patients were offered the same follow-up and support after discharge from the hospital on weekends. The staffing in home care services, nursing homes, and rehabilitation facilities, where patients are discharged to, is also reduced on weekends. Accordingly, there may be less time to capture any changes in the patients’ condition and medication list. Unfortunately, we had no information on the availability of specialist staffing in our study. Even emphasizing the importance of sufficient evaluation and follow-up for the frail hip fracture patients, we cannot, based on our results, determine whether this actually can be an explanation for the differences found.

We found that hospital LoS was significantly shorter for patients discharged on weekends (4 days) compared to weekdays (6 days) both when looking at the whole study population and when only looking at patients discharged to a nursing home. Rajamaki et al. [22] studied 12,532 hip fractures and found increased risk of readmission within 30 days after hospital discharge in patients with short (< 4 days) length of stay. This makes it difficult to determine whether the increased mortality observed in patients discharge during the weekend is due to the timing of discharge itself (weekend vs. weekday) or whether it is caused by shorter LoS for patients discharged during the weekend. According to our results, there is a need for increased attention and clear criteria that should apply for discharge both on weekdays and weekends, as well as regardless of the type of patients or the type of surgery.

No statistically significant nor clinically important difference in 30 day readmission risk between weekday and weekend discharge could be found in our study. This is in line with a previous study on hospital discharge following major surgeries such as total hip arthroplasty, abdominal aortic aneurysm repair, colectomy, pancreatectomy which reported no significant increased 30 day readmission risk after discharged on weekends compared to weekdays [23].

The large number of patients is the major strength of the present study. Another strength distinguishing this study from earlier studies is data on patient characteristics (age, sex, ASA class, and cognitive status). Further, this study includes data on discharge destination and readmissions. Finally, we present national results, increasing the external validity of the results.

The main limitation of an observational register-based study is that findings are less conclusive than those from a randomized clinical trial (RCT). The research questions at hand in this study are, however, not possible to investigate in an RCT, and an observational study is therefore the best approach. Despite adjusting for patient characteristics, fracture type and type of surgery, there is a risk for residual confounding when using registry data. Even if we had applied methods such as propensity score matching, matching could only have been performed based on available variables. Ideally, we would have had access to additional data, such as pre-fracture living situation and a more comprehensive comorbidity index, which could have improved confounder control. Due to the absence of such key information, we are not confident that a propensity score matching approach would have significantly altered the results or conclusions. Moreover, propensity score matching would have limited the number of included patients to the size of the smallest group. Further loss of data would likely occur in subgroup analyses. To preserve statistical power and generalizability, we therefore opted to include all patients with complete data and adjust for confounding using multivariable regression.

ASA classification and binary cognitive status represent limited proxies for assessing comorbidity and frailty and do not capture the full complexity of a patient’s frailty. The NHFR lacks additional variables that could have provided a more comprehensive evaluation of comorbidity beyond these two measures. Ideally, we would have included a more comprehensive comorbidity index, such as the Clinical Frailty Score or Charlson, alongside a validated cognitive assessment tool, like the Mini-Mental State Examination (MMSE). Despite the absence of these more comprehensive measures, our results remained mostly consistent when performing sub-analyses based on the available variables. Another limitation of the study is that nearly 50% of patients from the study period (2008–2018) could not be included due to missing information on discharge destination in the NPR or due to technical problems with identifying and linking of patients between the NPR and NHFR. Nevertheless, the dataset remains large and includes a substantial number of patients. Moreover, the inability to link data is not associated with any specific type of patient, which supports our assumption that the study population is broadly representative.

We had unfortunately, no information on pre-fracture living arrangements for the patients. Accordingly, we could not perform separate analyses for home-dwelling patients or long-term nursing home residents. In addition, due to inaccurate classification of discharge destination in the NPR, we were only able to perform sub-analyses on patients discharged to a nursing home facility. Finally, we lack information on important factors during discharge from the hospital, such as the availability of specialist staffing both at hospitals and at the discharge destination.

The results from our national registry study are influenced by the Norwegian Health system where most patients with hip fracture have a relatively short LoS and are discharged to nursing homes or rehabilitation facilities. This is also true for the Nordic and several European countries. Our findings may not be relevant for countries with long hospital LoS after surgery for hip fracture and where patients are not offered rehabilitation after discharge.

Further research into the discharge criteria used on weekdays and weekends, and further research that include more information about patients’ previous illness and their comorbidities would be valuable, as well as treatment after discharge from the hospital and patients’ ability to regain their previous functional level.

## Conclusion

Weekend discharge was associated with increased 30 day and 1-year mortality compared to weekday discharge. These findings were present regardless of patient characteristics (except patients aged 65–74 years) and for patients discharged to a nursing home. No statistically significant nor clinically important difference in 30-day readmission risk for weekend discharge compared to weekday discharge could be found, but there were minor differences for some subgroup of patients. Our findings indicate a need for increased attention and clear discharge criteria for the discharge of hip fracture patients also over the weekends.

## Data Availability

The data in the NHFR is stored in a secure server area and is accessible only to a limited number of persons working with the NHFR.

## Code availability

IMB-SPSS Statistics, version 29.0 for Windows (IBM Corp, Armonk, NY, USA), and the statistical package R, version 4.2.3 (http://www.R-project.org).
